# Synchrotron-generated microbeams induce hippocampal transections in rats

**DOI:** 10.1038/s41598-017-18000-x

**Published:** 2018-01-09

**Authors:** Erminia Fardone, Benoît Pouyatos, Elke Bräuer-Krisch, Stefan Bartzsch, Hervè Mathieu, Herwig Requardt, Domenico Bucci, Giacomo Barbone, Paola Coan, Giuseppe Battaglia, Geraldine Le Duc, Alberto Bravin, Pantaleo Romanelli

**Affiliations:** 1 European Synchrotron Radiation Facility (ESRF), Grenoble, France; 2 Grenoble Institut des Neurosciences, Inserm U836, Université Joseph Fourier, Grenoble, France; 3 Department of Radiation Oncology, Klinikum rechts der Isar, Technical University of Munich, Munich, Germany; 4 The Institute of Cancer Research, London, United Kingdom; 5 I.R.C.C.S. Neuromed, Pozzilli (IS), Italy; 6 Department of Physics, Ludwig Maximilians University, Garching, Germany; 7 Department of Clinical Radiology, Ludwig Maximilians University, Munich, Germany; 8 Brain Radiosurgery, Cyberknife Center, Centro Diagnostico Italiano (CDI), Milano, Italy; 9 Present Address: Department of Biological Science and Program in Neuroscience, Florida State University, Tallahassee, FL USA

## Abstract

Synchrotron-generated microplanar beams (microbeams) provide the most stereo-selective irradiation modality known today. This novel irradiation modality has been shown to control seizures originating from eloquent cortex causing no neurological deficit in experimental animals. To test the hypothesis that application of microbeams in the hippocampus, the most common source of refractory seizures, is safe and does not induce severe side effects, we used microbeams to induce transections to the hippocampus of healthy rats. An array of parallel microbeams carrying an incident dose of 600 Gy was delivered to the rat hippocampus. Immunohistochemistry of phosphorylated γ-H2AX showed cell death along the microbeam irradiation paths in rats 48 hours after irradiation. No evident behavioral or neurological deficits were observed during the 3-month period of observation. MR imaging showed no signs of radio-induced edema or radionecrosis 3 months after irradiation. Histological analysis showed a very well preserved hippocampal cytoarchitecture and confirmed the presence of clear-cut microscopic transections across the hippocampus. These data support the use of synchrotron-generated microbeams as a novel tool to slice the hippocampus of living rats in a minimally invasive way, providing (i) a novel experimental model to study hippocampal function and (ii) a new treatment tool for patients affected by refractory epilepsy induced by mesial temporal sclerosis.

## Introduction

Microscopic arrays of X-ray beams originating from a synchrotron source can induce the equivalent of a microsurgical neocortical or hippocampal incision by delivering very high doses of radiation to tissue slices of microscopic thickness. Neurons, glia and axons along the penetration path receive peak doses up to 1000Gy, and die, while the very adjacent tissue is exposed to much lower valley doses (less than 6Gy) unable to induce histologically evident tissue damage^1^. In essence, synchrotron-generated cortical transections provide a microradiosurgical equivalent of multiple subpial transections (MST), a non resective surgical technique developed to treat patients with medically-refractory epilepsy involving eloquent cortex^2–4^. This technique requires the placement of vertical incisions through the epileptic cortex in order to cut the horizontal axons responsible of the propagation of seizures while preserving the vertical axons subserving neurological functions. The vertical columns working as the basic unit of cortical function are disconnected but not injured by MST, allowing the treatment of epileptic foci located over sensorimotor or language cortex not amenable to surgical resection. Microbeam transections have been performed over an epileptogenic focus located in sensorimotor cortex, with almost immediate abolition of seizures and excellent preservation of motor function^5^. These results suggested further investigations to assess the potential of microbeam transections to modulate cortical and hippocampal functions and to treat focal epilepsy and other brain disorders as well as brain tumors. Further studies on this novel approach have also been encouraged by the ongoing development of devices delivering submillimetric beams able to generate the equivalent of a microbeam transections which could be available for clinical testing soon. Microbeam transections might add a powerful new tool to the clinical treatment of epilepsy and, more in general, to modulate cortical functions in a wide variety of neuropsychiatric disorders^5^. There is currently no equivalent to this technique either using radiation or microsurgery. Stereotactic radiosurgery, which is the most refined technique to deliver focal irradiation, cannot provide currently beams smaller than 4 mm and doses exceeding 100 Gy are often associated with severe side effects such as radionecrosis and massive brain edema. Microsurgery provides the ability to generate cortical transections of approximately 1 mm size but require a craniotomy and the manipulation of the cortex. None of the two allows to change the size of the transections, the distance between the transections, the location and extension of the cortical or hippocampal region transected, while this is possible with microbeam transections.

Stereotactic radiosurgery (SRS) provides today an attractive less invasive than traditional surgery approach to treat cortical, hippocampal or diencephalic epileptic foci but it is limited by the relatively low doses deliverable with current techniques, by the long delay needed to achieve seizure amelioration using currently allowed doses and by the side effects discussed above (severe radio-induced edema and radionecrosis)^6^.

As compared with SRS, synchrotron-generated X-ray microplanar beams (microbeams) provide a completely new tool to deliver extremely high doses of radiation restricted to microscopic volumes^1,5,7,8^. The dose spreading outside the beam path is minimal, allowing to release doses of several hundred Gray (Gy) to tissue slices of microscopic thickness. Outside the beam path, there is a brisk dose reduction: a few dozen of microns away from the beam the dose delivered to the tissue is already less than 5% than the in-beam dose^9^. This unique irradiation modality provides the ability to generate the equivalent of a microsurgical incision in a minimally invasive way. Synchrotron microbeams can be delivered with submillimetric precision over a fraction of a second to selected brain regions, thus ablating a tumor or an epileptic focus^1,5,7,8^. The European Synchrotron Radiation Facility (ESRF, Grenoble, France) has provided us a unique tool to treat experimental epilepsy by generating microbeam transections in eloquent brain regions^5^. We have explored the ability of microbeams to generate cortical and hippocampal transections by delivering peak doses ranging from 150 to 600 Gy to microscopic brain volumes of 75 to 600 µm in thickness. We have observed that spatially restricted microbeam irradiation, even delivered at high-dose, provides an exceptional degree of protection from radio-induced damage to neurons and glial cells adjacent to the microscopic slices of irradiated cortex and hippocampus. Microbeam transections have been generated within an epileptic focus located in eloquent cortex to test their ability to abolish seizures while preserving neurological function^5^. Seizure control was thus achieved in rats with status epilepticus generated by focal injection of kainic acid in the sensorimotor cortex. The sensorimotor cortex transections generated no motor deficits. This experimental work confirmed that microbeam transection can stop seizures while preserving the function of the irradiated cortex^10^. However, the origin of most cases of drug-refractory seizures in adult patients is the hippocampus vs. the neocortex. Accordingly, we tested the hypothesis that microbeams irradiation of the hippocampus is safe and does not induce severe side effects in healthy rats.

## Results

### Dosimetry

**Figure 1:**
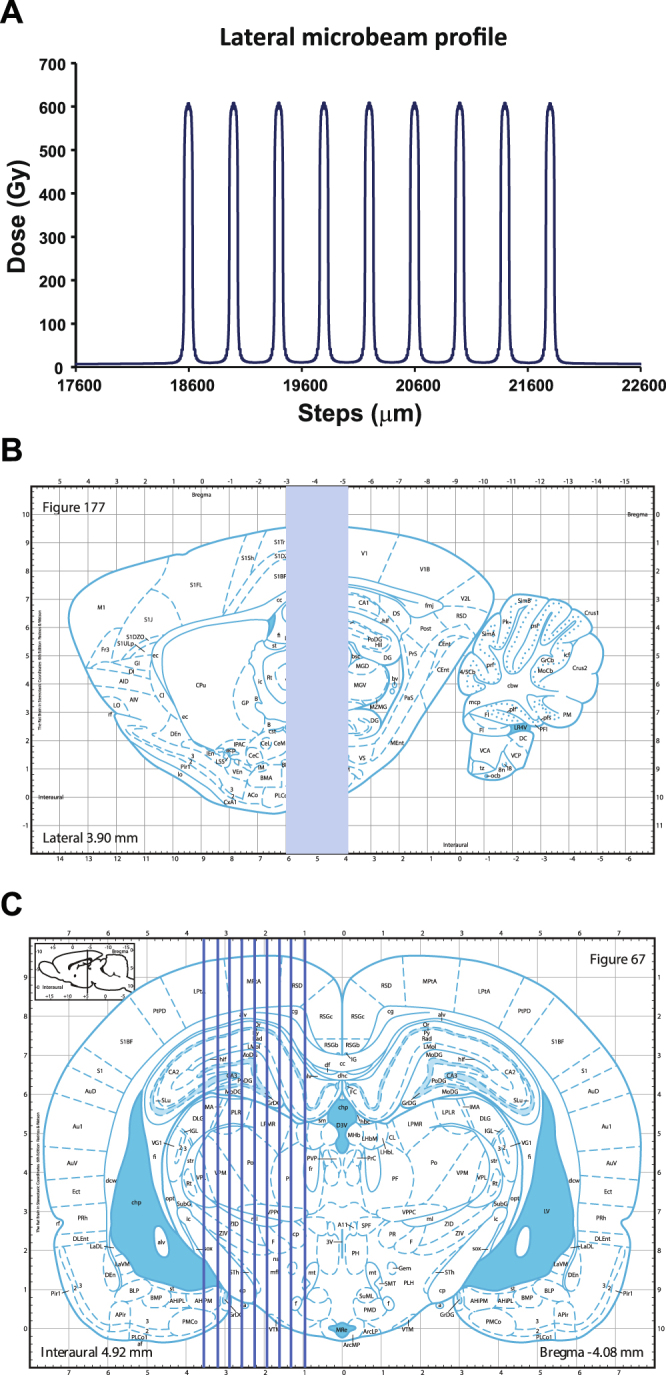
Microbeam dosimetry and schematic representation of irradiation geometry. (**A**) Plot of the lateral dose profile in water. (**B**) Sagittal section of the Paxinos and Watson’s rat brain atlas^30^ showing the geometry of microbeam irradiation. Coronal section of the left hippocampus irradiation is shown in (**C**). The irradiation of the right hippocampus is specular to (**C**). Arrays of 75 μm thick microbeams (9 beams, center-to-center spacing: 400 μm) were delivered perpendicular to the dorsal hippocampus (from −3 mm to −5 mm posterior to the bregma) using an atlas-based image guided X-ray setup.

**Figure 2:**
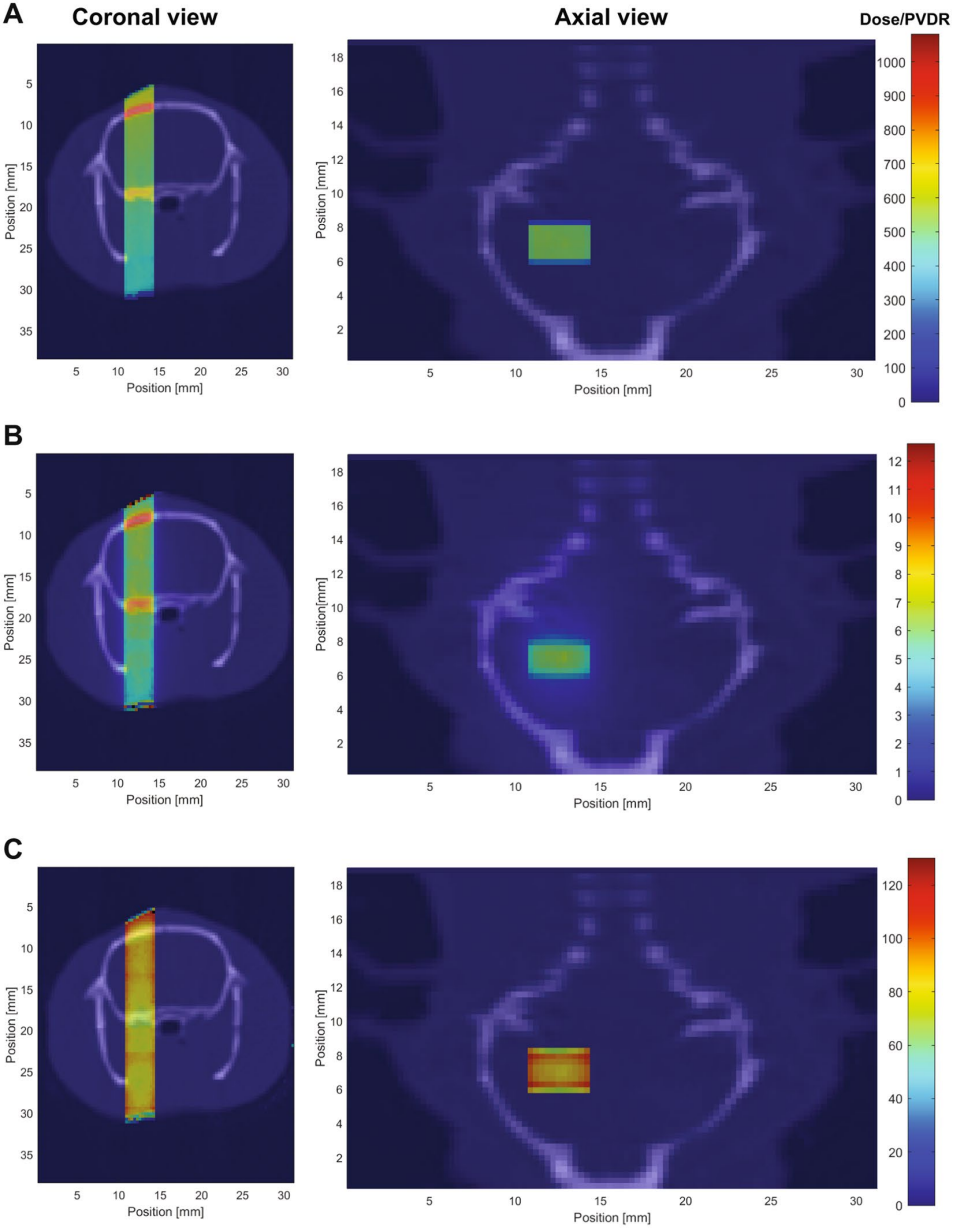
Coronal and axial views of the rat showing peak dose (**A**), valley dose (**B**) and peak to valley dose ratios (PVDR) **(C)**. Doses are normalized such that the peak dose at 3 mm depth in a homogeneous water phantom would be 600 Gy. The microbeam field contains 9 parallel 75 µm wide and 2 mm high microbeams with a center-to-center spacing of 400 µm. The peak dose is between 520 and 590 Gy in the brain, depending on the distance from the beam entrance; the valley dose ranges between 6.4 and 6.8 Gy. The PVDR measures between 80 and 87. The figures overlay Hounsfield units of a computed tomography image acquired at the ESRF at 35 keV (in gray) and doses using the color scale on the right.

The dose profile into the target consists of high doses along the microbeam path (peak) and low doses in the spaces between them (valley) (Fig. 1A). A measured dose profile in homogeneous water is visible in Fig. 1A; the profile was acquired following the protocol described in Bräuer-Krisch *et al*.^11^. The microbeam field contains 9 parallel 75 µm wide and 2 mm high microbeams with a center-to-center spacing of 400 µm, delivered following the coordinated reported in the Methods section and illustrated in Fig. 1B and C. In MRT, the reference valley dose is normally assumed as the minimum dose in between two microbeams. Figure 2A shows peak to valley dose ratios (PVDR), Fig. 2B peak and Fig. 2C valley dose on respectively axial and coronal views. The dose is normalized such that the peak dose would be 600 Gy at 3 mm depth in a homogeneous water phantom. The peak dose is between 520 and 590 Gy in the brain, depending on the distance from the beam entrance; the valley dose ranges between 6.4 and 6.8 Gy. The PVDR measures between 80 and 87.

### Early Immunohistochemistry

**Figure 3:**
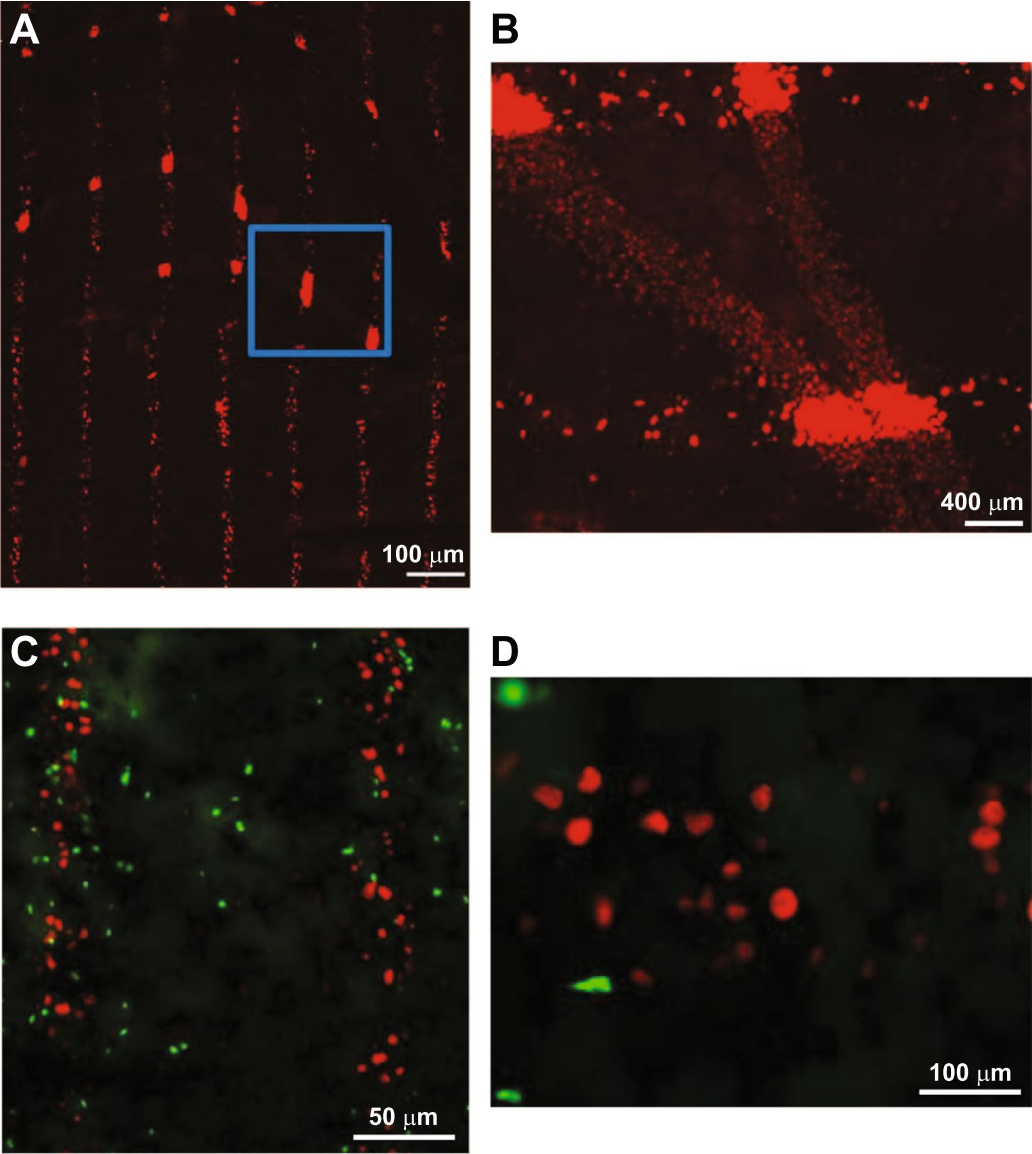
Microbeams induce early cell death. Immunohistochemistry of phosphorylated γ-H2AX-positive cells (in red) and Ki67-positive cells (in green) in the rat dorsal hippocampus 48 hours after microbeam irradiation (**A**). Magnification of the square in A (**B**). Ki67-positive cells are visible within and between the microbeam path (**C**). Higher magnification of C (**D**).

Immunohistochemistry of phosphorylated γ-H2AX showed a well-defined column of cell death along the microbeam penetration pathway 48 hours after irradiation. There was a brisk transition from the nuclei of cells positive for phosphorylated γ-H2AX, a histone modification in response to DNA double-strand break, which occurs within minutes of break induction, and is a sign of dying cells along the beam path. Figure 3 shows a bright red color for phosphorylated γ-H2AX, and the nuclei of viable cells just a few microns away. Inside and near the beam path, we observed early cell proliferation, as detected by immunohistochemistry of Ki67, a marker of proliferating cells. Ki67-positive cells were visible as green-colored nuclei (Fig. 3) 48 hours after irradiation. The characterization of these cells requires further studies.

### Behavioral observations

All rats tolerated very well the procedure. Two animals were randomly chosen and sacrificed within 48 hours from irradiation to verify the immediate effects of microbeam transections. The remaining 8 rats survived and gained weight regularly during the observation period of 3 months. No sign of behavioral abnormalities or neurological disorders was observed during weekly observations.

### MR imaging

**Figure 4:**
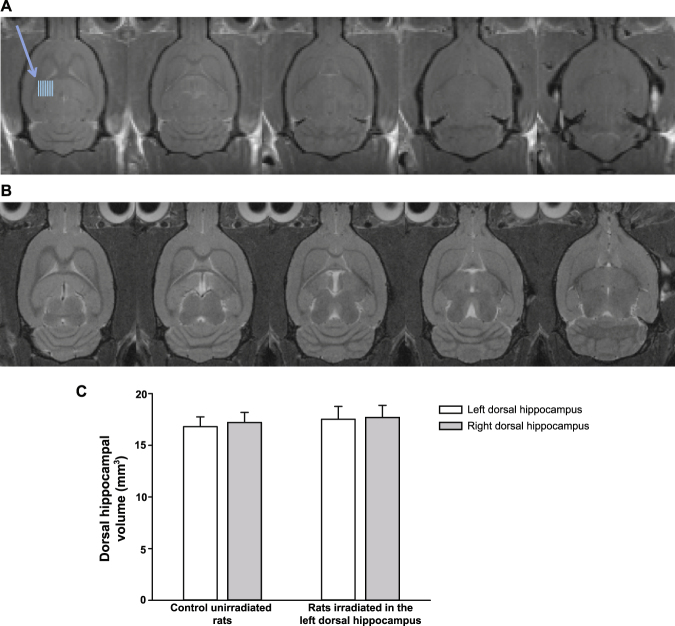
MRI does not show any sign of radionecrosis or radio-induced edema. Representative T1-weighted post-contrast (**A**) and T2-weighted (**B**) MRI performed 3 months after irradiation of the left dorsal hippocampus are shown. Blue lines indicate the irradiation zone in the left dorsal hippocampus. Note the absence of any sign of brain damage induced by the microbeams (peak dose: 600 Gy). Quantification of left and right dorsal hippocampal volumes in control unirradiated rats and in irradiated rats. Values were calculated directly from the DICOM images and are the means ±  SD (n = 2–4). Statistical analysis was performed by Two-Way ANOVA (p = 0.992, irradiation x brain area) (**C**).

No sign of irradiation was observed either on T1 post-contrast and T2 imaging carried out 3 months after microbeam irradiation. T1 post-contrast images show no signal changes and no extravasation of contrast medium in the irradiated regions. No sign of radionecrosis was visible (Fig. 4A) and T2 post-contrast images were remarkable for the absence of hyperintense signal suggestive of radio-induced hippocampal edema (Fig. 4B). Hippocampal volumes and shapes are similar on the irradiated and non-irradiated side. Dorsal hippocampal volumes in irradiated vs. non-irradiated (control) sides were compatible (Fig. 4C).

### Histology

**Figure 5:**
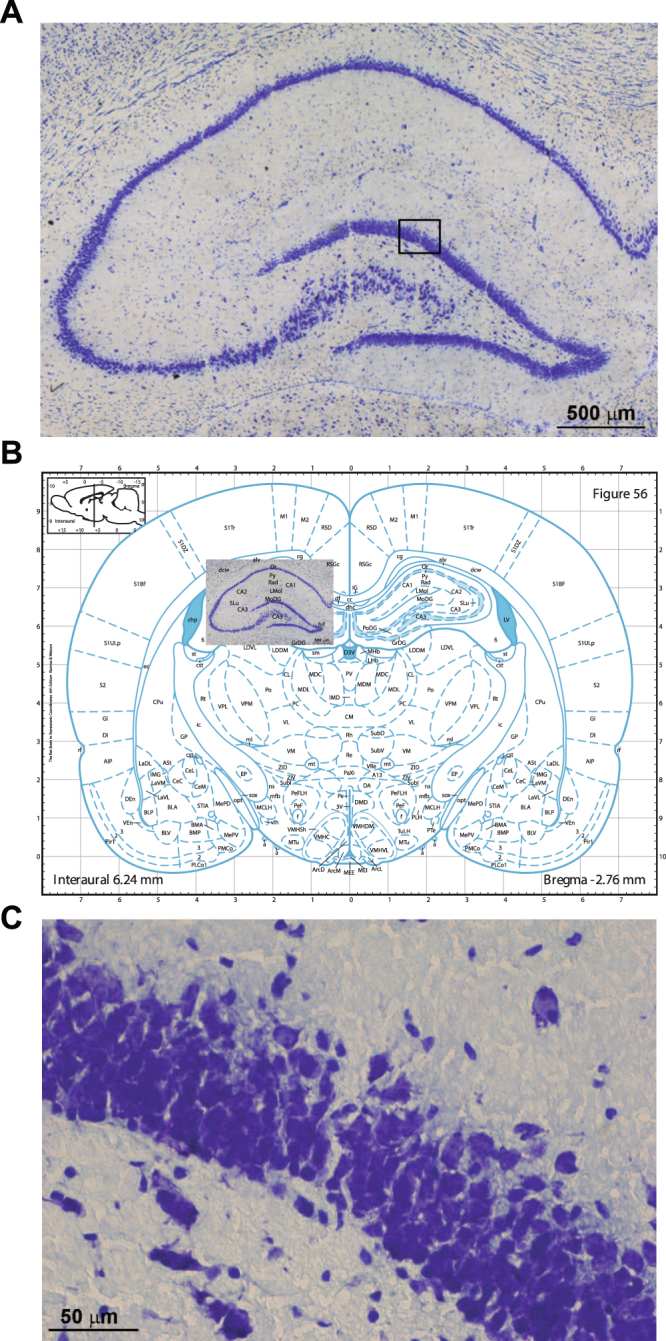
Histological analysis of rat hippocampus 3 months after microbeam-induced transections. Clear-cut transections through the hippocampus are visible with no evident collateral damage (**A**). Overlay of the histology over the equivalent slice taken from the Paxinos and Watson rat brain atlas. The hippocampal architecture is very well preserved (**B**). The square in the dentate gyrus in (**A)** is highlighted at higher magnification in (**C**). Note that the hippocampal layers near to the transections are not affected.

Histological analysis carried out 3 months after microbeam irradiation showed clear paths of cell death corresponding to the beam penetration pathway (Fig. 5A). These transections were characterized by sharp margins. Shape and volume of the transected hippocampus and dentate gyrus have normal appearance (Fig. 5B). The loss of cells along the irradiation path is almost completely “rescued” as observed at high magnification, where normal neurons are visible immediately outside the beam path (Fig. 5C). There is only the presence of these transections due to cell loss.

## Discussion

Focal irradiation of an epileptic focus through SRS is an emerging treatment for medically refractory seizures, providing a non-surgical approach which is mainly limited by the delay of efficacy (several months are typically required to obtain seizure relief) and side-effects (long-lasting radio-induced edema requiring prolonged steroid administration)^11,12^. Current LINAC- or cobalt-based technologies do not allow to deliver high doses to cortical slices of millimetric size, thus replicating the exquisite cortical incisions generated by MST. A novel exciting approach combining the advantages of SRS and MST has been investigated at the ESRF, where arrays of microplanar beams have been used to generate cortical transections equivalent to MST in a minimally invasive, bloodless way.

Romanelli and Bravin^1^ hypothesized that microbeam cortical transections may be explored as a way to modulate cortical function without injury to the columnar organization. This technique would have preserved the vertical axons and transected horizontal seizure-spreading axons, determining an effective way to parcellize a neocortical epileptic focus and stop seizure propagation while preserving cortical function. Microbeam transections of the sensorimotor cortex were then demonstrated to reduce seizure in an epilepsy rat model^5,8^ and video-EEG analysis showed abolition of seizures after the microbeam delivery^13^. Microbeam transections applied to a rat model of absence epilepsy (GAERS rats) also altered the abnormal neuronal activities for at least 9 weeks^14^.

Here, we show that the dorsal hippocampus was transected with microbeams, where the average dose given to the 75 µm column of tissue crossed by a microbeam (peak dose) was >80 times higher than the dose in the tissue placed between the paths (valley dose) (see Fig. 2). Neuronal cells, glial cells and axons along the penetration path are ablated without damage to adjacent cells, thus originating the equivalent of a microsurgical incision. Therefore, synchrotron-generated cortical transections provide a microradiosurgical equivalent of MST, which, however, requires an invasive procedure involving all the risks related to open surgery. Microbeam transections are performed in a minimally invasive way and the size and spacing of the transections can be modified by the surgeon according to the needs. The histological effects of hippocampal transection have been investigated here in healthy rats showing no MR signal changes, radioinduced edema and radionecrosis, and excellent preservation of the hippocampal architecture after 3 months. Immunohistochemistry performed immediately after the irradiation showed immediate cell death along the microbeam path with proliferative response within the path itself and in the immediately adjacent tissue. This observation shows that a high-dose (in the range of hundreds of Gy) stereoselective irradiation restricted to volumes of microscopic size allows cell replication over the immediately adjacent tissue, visible after 48 hours. The marker used here (Ki67) does not allow to verify the glial versus neuronal origin of the replicating cells but the induction of neural progenitor proliferation cannot be excluded and we are working to further characterize this proliferative response. In humans, radio-induced edema and radionecrosis develop 6 to 12 months after irradiation (even if late radionecrosis is possible, especially with conventional radiotherapy). An experimental study to assess toxicity on primates of conventional irradiation (total dose: 40 Gy delivered in 8 fractions, of 5 Gy each, over one month) lead to the sacrifice of all the animals 6 months after the irradiation due to severe consequences of edema and radionecrosis^15^. Considering the exceptionally high doses used here (600 Gy delivered in 0.17 s), we observed parallel cuts immediately and 3 months after irradiation through the hippocampus, providing an “elegant” slicing of the region. After 3 months, MRI and conventional histology confirmed the total absence of radio-induced edema and/or radionecrosis. On the contrary, studies on hippocampal irradiation using conventional stereotactic radiosurgery after lower doses have shown extensive MRI and histological changes with massive radionecrosis and extensive radio-induced edema at this time point. In another study^16^, rats received stereotactic unilateral hippocampal irradiation with doses ranging from 5 to 130 Gy and evaluation by T2 MRI showed evidence of radio-induced edema and radionecrosis in rats receiving 90 and 130 Gy at 3 months. Mori *et al*.^17^ have performed MRI with T1- and T2-weighted series in rats 7, 21 and 42 days after hippocampal injection of kainic acid followed by stereotactic radiosurgery delivering doses of 20, 40, 60 and 100 Gy. Hippocampal injury related to kainic acid injection was found in all cases, but evidence of additional radionecrosis was found in rats irradiated with 100 Gy. Finally, Ishikawa *et al*.^18^ have shown widespread radio-induced edema (visible on T1 and T2-weighted MRI performed 2 weeks after irradiation) in rats receiving 200 Gy and sacrificed after 2 weeks. In rats receiving lower doses (100, 75, 50, 25 Gy), MRI was done sequentially every two weeks up to 16 weeks when rats were sacrificed for histological analysis. Evidence of radionecrosis and edema were found on MRI in 200 Gy irradiated rats extending along the white matter to the ipsilateral thalamus, hypothalamus, striatum, and to the contralateral external capsule. Similar findings developed 8–10 weeks after irradiation with 100 Gy. Rats receiving 75, 50 and 25 Gy developed less striking changes but MRI was still remarkable for radionecrosis and radio-induced edema, confirmed by histological analysis carried out 4 months after irradiation. Considering that maximum dose delivered to the hippocampus before our study was 200 Gy (causing damage to induce the sacrifice of rats after 2 weeks) and based on the observations of several reports^19–22^ delivering stereotactic irradiation to the hippocampus, we set our observation time at 3 months. Our findings are very encouraging having found no damage outside the transection 3 months after the delivery of a microbeam array carrying an incident dose of 600 Gy with a valley dose of 6.4 Gy. We are now studying whether transections can be obtained with lower doses (150 or 300 Gy), thus lowering the valley doses to levels fully compatible with those of human radiation therapy.

In conclusion, our preliminary results are encouraging, suggesting that microbeam transections hold a great potential to modulate brain function and to treat focal epilepsy by mimicking MST in a minimally invasive way without carrying out surgery. Moreover, as previously shown^5^, seizure control and preservation of motor function after eloquent cortex transections provide the groundwork for an exciting new application of synchrotron-generated microbeams, opening the way to the application of this new concept to the most common source of medically-refractory seizures, the hippocampus, responsible of mesiotemporal epilepsy. Therefore, the development of novel devices delivering submillimetric beams might add a new powerful tool to the clinical treatment of epilepsy and, more in general, to modulate cortical and hippocampal functions in a wide variety of neuropsychiatric disorders.

## Methods

### Animals

Male Wistar rats (175–200 g) were purchased from Charles River Laboratories (L’Arbresle, France) and were maintained under controlled environmental conditions (temperature: 22 ± 2 °C, humidity: 40–60%) on a 12-hour light/dark cycle with food and water *ad libitum*.

All experimental protocols related to animal care strictly conformed to the Guidelines of the French Government and the 2010/63/UE directive and were approved by the ethical committee of the European Synchrotron Radiation Facility, Grenoble. All efforts were made to minimize the potential sufferance and discomfort of animals and their number.

### Irradiation parameters

All irradiations were carried out at the ID17 Biomedical Beamline of the ESRF. X-rays are emitted by the wiggler source located in the straight section of the storage ring. The wiggler produces a continuous (white) beam spectrum filtered for this study by a succession of five attenuators [Be (0.5 mm), C (1.5 mm), Al (1.5 mm), Cu (1.0 mm) and Al (1.5 mm)] resulting in a photon spectrum extending from about 50 to 350 keV, with a mean energy of approximately 105 keV^10^. The quasi-laminar beam is spatially fractionated into an array of microbeams of variable size by using an adjustable multislit collimator^23^. The X-ray fluence produced by the wiggler determined an entrance dose rate of around 14,000 Gy/s in the homogeneous 2 × 2 cm^2^ field, allowing the deposition in a fraction of second of doses of hundreds of Grays along the microscopic planes.

Animals were irradiated with a microbeam array made by 9 parallel microbeams of 75 µm in width (as full width at half maximum) and 400 µm center-to-center distance. Before irradiation, each microbeam was individually measured using a ~1–1.5 µm wide tungsten aperture moved in front of it; the signal was then recorded by an ionization chamber. The microbeam size was highly homogeneous and a maximum difference of ±1 µm was measured between the beams thanks to the regular geometry of the collimator.

The irradiation target was identified on low dose (<5 mGy) radiographies performed using a highly attenuated beam. High resolution images (22 μm pixel size) were acquired by a Frelon camera-based detection system^24^ using the propagation-based phase contrast technique^25^. This method allowed a precise determination of the bregma (uncertainly: < 0.2 mm), which was the origin of the rat coordinates.

### Monte Carlo dosimetry simulation

The dose distribution was calculated using the Monte Carlo toolkit Geant4, version 10.2.2. based on a CT of a rat acquired at 35 keV at the synchrotron. Hounsfield units were translated into material composition and density using the method of Schneider *et al*.^26^. For the spectrum at the ESRF photoelectric effect, Compton scattering and Rayleigh scattering are relevant and taken into account. Cut-off ranges for electrons were set to 1 µm. Energy absorption was scored independently for peak and valley on grid defined by the CT cube^27^. Valley dose is defined as average dose in the central 60% (195 µm) of the valley and peak dose as average dose in the central 80% (60 µm) of the peak. A total number of 2^.^10^9^ primary photons were simulated according to a phase space model that incorporates polarization, collimator absorption and leakage radiation^28^.

### Irradiation of rats

Rats were anesthetized with an intraperitoneal injection of xylazine/ketamine (64.5/5.4 mg*kg^−1^) for unilateral irradiation of the dorsal hippocampus using a microbeam array. Five animals received right hippocampal transections and 5 animals underwent left hippocampal transections. Anesthetized rats were placed in vertical prone position on a custom-made stereotactic frame fixed on the Kappa goniometer (Huber, Germany), which allows to translate and rotate the animal in front of the fixed horizontal X-ray beam^29^. The spatial configuration of microbeams was checked by Gafchromic^®^ film. The left dorsal hippocampus received a microbeam array measuring 2 mm in the antero-posterior (AP) direction (3 to 5 mm posterior to the bregma) and 3.6 mm in the mediolateral (ML) direction (−1 to −4.3 mm lateral to the midline)^30^. The right hippocampus received an identical irradiation (coordinates: AP: 3 to 5 mm posterior to bregma; ML: 1 to 4.3 lateral to midline).

### Behavior assessment

Rats were weighed weekly and observed daily to evaluate the development of behavioral and physiological abnormalities and signs of pain and distress (e.g. weight loss, aggressiveness, apathy, hemiparesis, lethargy).

### Magnetic Resonance Imaging (MRI) acquisition

Magnetic resonance imaging (MRI) was performed at the MRI platform of the Grenoble Institute of Neurosciences (Grenoble, France) 3 months after irradiation on 2 non-irradiated rats and 4 irradiated rats. Rats were anesthetized by isoflurane (5% for induction and 2.5% for maintenance) for MRI studies. Images were acquired using a 7 T Bruker Avance III system using a quadrature volume coil. A T2-weighted sequence (acquisition parameters, according to Serduc *et al*.^31^, were TR: 4000 ms, effective TE: 33 ms, FOV: 30 × 30 mm^2^, matrix: 256 × 256, slice thickness: 0.5 mm, N average = 2, duration 4 min 16 s) was acquired to visualize the hippocampal anatomy bilaterally and check for radio-induced edema and/or radionecrosis. A T1-weighted post-contrast sequence (acquisition parameters, according to Pouyatos *et al*.^14^, were TR: 1300 ms, effective TE: 7.7 ms, FOV: 30 × 30 mm^2^, matrix: 256 × 256, slice thickness: 0.5 mm, N average = 4, duration 4 min 4 s) was then acquired 5 min after an intravenous injection of Gd-DOTA (200 µmol*kg^−1^, Dotarem, Guebert^®^, France) to verify evidence of contrast extravasation following radiation damage of the blood-brain barrier.

### Image processing

The dorsal hippocampal volumes were computed from the DICOM images by manually highlighting the target area multiplied by slice thickness using the MicroDicom viewer software (www.microdicom.com).

### Immunohistochemistry and histology

Forty-eight hours or 3 months after irradiation rats were killed by intraperitoneal administration of a Dolethal^®^ overdose. Brains were dissected out and immediately frozen at −20 °C or fixed in 4% paraformaldeide (PFA). Fifteen µm thin coronal brain sections were cut at −22 °C on a cryostat and stained with thionin (Nissl staining). For immunohistochemistry, 15 µm thin coronal brain sections were cut on a microtome. Slices were blocked with donkey normal serum (DNS, Interchim) in phosphate-buffered saline (PBS) 1X for one hour (PBS/DNS 5%). Primary antibodies were anti-phosphorylated γ-H2AX (1/500, 05636, Upstate Biotechnology, Lake Placid, NY) and Ki67 (1/200, Clone S6, Lab Vision Corporation, Fremont, CA) diluted in PBS/NDS 1%. Sections were washed 4 times with PBS, then incubated with the secondary antibodies Alexa fluor-conjugated donkey F(ab’)2 (1/200, #A31571 and #A11056, Invitrogen, Carlsbad, CA) for 2 h at room temperature. Sections were examined with a Nikon Eclipse E600 microscope equipped for epifluorescence.

### Statistical analysis

Data were analyzed by Two-Way ANOVA.
